# Undulatory Hypothesis Applied to Sensation

**Published:** 1871-11

**Authors:** 


### Undulatory Hypothesis Applied to Sensation.

In the course of the discussion upon a paper read by Dr. Brown-Sequard, at the late meeting of the British Association, Dr. McDonald, F. R. S., of Dublin, said that he had long felt some difficulty about adopting the hypothesis that there existed distinct conductors for various sensations, as those of heat, pain, tickling, contact, &c. In explanation of the remarkable cases sometimes met with, in which an individual who felt perfectly the contact of one's hand, yet could not distinguish heat or cold, he proposed another. His hypothesic was, in fact, an application of the undulatory hypothesis to the propogation of nervous sensations; he supposed that sensations such as those of heat, pain, contract, as well as those of various colors, or form, of sound, were waves of different wave lengths; and that, under certain circumstances, some waves were absorbed or intercepted, while others passed on to the sensorium. He drew an analogy or illustration of his hypothesis from Prof. Tyndall's well-known experiments on the absorption of radiant heat by vapors or scents passed into the air filling a glass tube. The glass tube in this experiment represented the nerve tubule, the slight change effected in the air contained within it, produced by the introduction of the minutest quantity of scent, causes an absorption *in transitu* of some waves of heat; others pass. The experiment of seeing the complementary color upon gazing at a white ground, after looking upon a colored disc, might be explained thus: A single chemical change is effected in the nerve tubule by gazing at the colored disc; when the white ground is looked upon, all undulations pass through *save* those which are absorbed, viz., those of the color previously looked at. This, of course, gives the complementary color. Many phenomena connected with sensation, Dr. McDonald conceived, would find in this hypothesis a simpler explanation than in that of distinct conductors.—*Chemical Gazette.* —*Baltimore Med. Journal.*

